# Bridging Sex and Gender in Neuroscience by Shedding *a priori* Assumptions of Causality

**DOI:** 10.3389/fnins.2019.00475

**Published:** 2019-05-09

**Authors:** Melissa M. Holmes, D. Ashley Monks

**Affiliations:** ^1^Department of Psychology University of Toronto Mississauga, Mississauga, ON, Canada; ^2^Department of Cell & Systems Biology University of Toronto, Toronto, ON, Canada; ^3^Department of Ecology & Evolutionary Biology University of Toronto, Toronto, ON, Canada

**Keywords:** behavioral ecology, definition, gender, polymorphism, sex

“The first task of every science is the clear definition of the object it has to investigate. In no science, however, is this preliminary task so difficult as in psychology; and this circumstance is the more remarkable since logic, the science of defining, is itself a part of psychology. When we compare all that has been said by the most distinguished philosophers and scientists of all ages on the fundamental idea of psychology, we find ourselves in a perfect chaos of contradictory notions.”

From Haeckel (1905)

We agree with Haeckel that definitional issues are paramount in science and that we should be mindful of our human biases, especially when thinking about subjects such as sex and gender that are so central to our identity. It is our view that sex and gender can both be incorporated into neuroscience research in a meaningful, rigorous way but not with the current dichotomous approach: sex and gender are not distinct variables with mutually exclusive causes and cannot be considered as such. Our use of the terms sex and gender herein will reflect current conventional definitions, including those associated with this special issue, with “sex” referring to biological attributes and “gender” referring to social structure and socially constructed roles, behaviors, and identities. Here we argue that the most significant restriction for the successful widespread inclusion of sex and gender into neuroscience research is definitional. We believe a comparative and interdisciplinary approach to this question will help desegregate current definitions and drive sex and gender science forward in an efficient, integrative, and conceptually accurate manner.

The definitional issues surrounding sex and gender originate in part from a conflation of observable traits with inferences as to their causality. That is, “biological” and “social” are attached to “sex” and “gender,” respectively in a familiar dialectic that echoes notions of nature and nurture and by extension determinism and free will. This conflation is problematic not only because it leads to conceptual ambiguity and fuels unnecessary disagreements (see Griffiths, 2002, for relevant discussion of “innateness”), but also because defining traits based on presumed causality introduces a major obstacle to scientific investigation. Our thinking and experimentation should not be constrained by definitions that on the one hand are difficult to observe (i.e., to categorize a trait as a manifestation of sex or gender requires knowledge of its cause) and on the other hand precludes causal investigation (traits thus defined are then canonized). Case in point is the assumption or assertion that gender is a non-biological, social construction, or that sex has a purely biological basis. This dichotomous causal inference implies orthogonality and is dubious even when only considering traditional laboratory rodents and humans, but all the more so when we take a truly comparative perspective.

As a species, we seem to cherish a belief that humans are fundamentally unique among animals, beyond the obvious fact that, by definition, all species are unique from each other. It is common even among academics to hold the view that humans have categorically unique cognitive and social abilities, such as language, self-awareness, technology, and culture. These beliefs persist despite evidence for at least rudimentary forms of these abilities in other species. Notable for the current discussion, there is compelling evidence for at least limited theory of mind (i.e., the ability to recognize mental states in others such as their goals, intentions, perceptions, knowledge, and/or beliefs) in diverse non-human animals. For example, chimpanzees (*Pan troglodytes*) are able to “pass” tests of theory of mind that are applied to human infants. These include: adaptively modifying food begging according to whether an experimenter is unable or unwilling to give food, correctly producing an action that was unsuccessfully attempted by an experimenter, using stealth adaptively to disguise food retrieval from a competitor, and selectively retrieving food that is unknown to competitor (reviewed in Call and Tomasello, 2008). As further example, western scrub-jays (*Aphelocoma californica*) not only have effective food caching strategies to minimize thieving from competitors, but also move the location of their caches when they are observed if they have prior experience thieving from others (reviewed in Clayton et al., 2007). Several rodent species also show evidence of self-other awareness in the form of empathy or consolation behavior (Burkett et al., 2016; Mogil, 2019). Whereas it is important to acknowledge doubt concerning the extent to which the adaptive performance of other animals or even human infants reflects true understanding of the mental states of others rather than resulting from more simple behavioral rules (see Penn and Povinelli, 2007), we believe it is fair to say diverse species have remarkably sophisticated social behavior, enabling behavioral responses that adapt to mental states of conspecifics.

If other animals can, for lack of a better word, understand the goals, intentions, perceptions, and knowledge of their conspecifics, why would we assume that they are incapable of having awareness of their sex or sexuality, or for these to be separate from their social environment? To the contrary, it seems to us that it would be remarkable if this did not occur to varying extents in non-human animals, and there is considerable evidence to support this conclusion. Other species, too, appear to make at least rudimentary assumptions about how conspecifics will behave based on biological and social cues and shift their sociosexual phenotype based on social environment. For example, in bluehead wrasse (*Thalassoma bifasciatum*), adult females can undergo complete sex change based on social cues, becoming sperm-producing males if the existing dominant male is removed from their habitat (Warner and Swearer, 1991). Furthermore, male *Astatotilapia burtoni*, a species of cichlid, exist in two morphs: territorial, aggressive males have striking coloration while non-territorial, subordinate males do not. Importantly, these morphs are plastic. Males can shift between phenotypes, showing changes in behavior, morphology, and neuroendocrinology, depending on their social environment (reviewed in Maruska and Fernald, 2018). Are these male morphs equivalent to different genders? We believe they could be considered as such, however gender is, by most current definitions, a manifestation of human sociocultural factors and is therefore exclusively applied to humans.

These comparative examples highlight that the intersection of numerous physiological and behavioral traits can manifest in predictable morphs beyond “male” vs. “female”. In behavioral ecology, polymorphism is defined as the occurrence of two or more forms/morphs/phenotypes at the same ontogenetic stage within a population. These morphs must be discontinuous and occur at a frequency higher than explained by the rate of mutation. Importantly, while the morphs themselves are discontinuous, trait expression can be either categorical or continuously distributed. For example, male plainfin midshipman fish (*Porichthys notatus*) exist in two morphs (reviewed in Forlano et al., 2015). Type I males are larger, establish and defend territories, and produce sonic vocalizations to attract females. Type II males are smaller, do not maintain territories, and do not produce the same vocal repertoire. Rather, they mate cryptically by ejaculating when females are laying eggs in the territory of a Type I male. In this species, the presence or absence of testes is categorical between males and females but is continuous within males with Type II males having a higher gonadosomatic index than Type I males, on average. Furthermore, polymorphisms can be strictly genetic or environmentally-cued. In sexually reproducing species, the most obvious example of a polymorphism—and this is not a coincidence—is the differentiation of an embryo along male or female lines. In mammals, this is a classic example of genetic polymorphism whereby the mechanism of sexual differentiation is provided by polymorphic sex chromosome genes (reviewed in Arnold et al., 2012). In several species, however, sex determination is environmentally controlled. For example, in leopard geckos (*Eublepharis macularius*), the gonadal sex of the individual is attributable to the temperature in which the egg incubates (Viets et al., 1993). This is not to say that environmentally-cued polymorphism is independent of genetics. Rather, while genetic polymorphisms result from discontinuously distributed but continuously active genetic material, environmentally-cued polymorphisms stem from universally distributed but differentially active genetic material (Clark, 1976). Key to the current debate, because polymorphism means “many forms,” it is an appropriate term for observable differences in form between members of a population regardless of how many forms and *whether or not the mechanism of morph determination is known* (Clark, 1976). Importantly, this concept inherently acknowledges the intersectionality of biological and environmental mechanisms.

Discussion about the intersections between genes and environment in the evolution of human sex and gender differences is ongoing (e.g., Smuts, 1995; Eagly and Wood, 2013; Liesen, 2013; Neuberg and Sng, 2013; Barker, 2015) and it has been argued that the social environment and/or culture are not entirely distinct from genetic and epigenetic mechanisms (see for example Jablonka and Lamb, 2014; Fine et al., 2017). Indeed, others have advocated for a redefinition and expansion of sex and gender categories (e.g., Fausto-Sterling, 2000; Fine, 2010; Jordan-Young, 2010; Hyde et al., 2019) or suggested methodological approaches to better integrate sex and gender in neuroscience research (e.g., Rippon et al., 2014; Joel and McCarthy, 2017; Hyde et al., 2019). However, some of this discourse is inherently based on dichotomous definitions of sex and gender whereas we further the call for an empirical, theoretically agnostic approach to the re-examination of sex and gender categories on the basis of observable traits in the absence of causal assumptions. Using polymorphism as a theoretical framework for all sex and gender research (both human and non-human animal), we can statistically determine if a given phenotype (behavioral, morphological, or otherwise) is a continuous or categorical variable and how different variables cluster together (or not). We can then analyze sex and gender variables using multilayer network analysis, which is specifically designed to explore multifaceted systems (e.g., Finn et al., 2019). We can incorporate chromosomal, hormonal, and morphological variables with key environmental variables including social and sexual experience, social rank, and current and evolutionary social/sociocultural milieu. We can disrupt networks *in silico* to identify putative causal relationships and generate testable hypotheses concerning orthogonality of the variables that define morphs. We have little doubt that some of these variables will cluster together and influence each other in clear and predictable ways, particularly given well-established links between chromosomes, gonads, and morphology. However, exactly how this happens will differ according to species and, importantly, a broad comparative approach will allow us to identify opportunities for modeling specific target mechanisms that might better align with the human condition.

In sum, we agree with the idea that neuroscientists should theoretically be both “sex-informed” and “gender-informed” but we do not think the current definitions of sex and gender facilitate this goal. We argue for a reevaluation of the current consensus definitions that primarily serve to dichotomize the biological and the social when these are inextricably intertwined in any social animal. As a result, these definitions serve to inhibit investigations of mechanism, broadly defined. Furthermore, it is our opinion that applying “sex” to non-human animals but both “sex” and “gender” to humans is fundamentally inaccurate and imposes further bias on the study of mechanism. To correct this, we either need to redefine gender to focus exclusively on those features that are *truly* unique to humans, which will require significant introspection and debate, or we need to more broadly apply gender concepts to non-human social animals. In pursuit of a desirable social goal (i.e., inclusion and equal opportunity for individuals) we should not ignore or deny the biological variability that exists and the mechanistic determinants that cause the variability. That is, variability is not solely caused by disadvantage, suppression, and prejudice. Conversely, in pursuit of a standardized, reductionist translational approach, we cannot ignore or deny species-specific social adaptations and the importance of social interactions on physiology. We fully acknowledge the complexity of studying/modeling sex and gender (e.g., Jordan-Young and Rumiati, 2012; Eliot and Richardson, 2016) but we believe this should be a source of scientific inspiration. We need to keep asking the questions, we just need to reframe how we do so. By taking a step back, shedding our biases about causation, and appreciating variability within and across species, we can revisit the consensus definitions of sex and gender in an unbiased, data driven way. We believe this will reframe how we study sex and gender and ultimately better reveal the interplay between an organism's brain, body, behavior, and environment.

## Author Contributions

All authors listed have made a substantial, direct and intellectual contribution to the work, and approved it for publication.

### Conflict of Interest Statement

The authors declare that the research was conducted in the absence of any commercial or financial relationships that could be construed as a potential conflict of interest.
